# Enhancing access to the Bibliome: the TREC 2004 Genomics Track

**DOI:** 10.1186/1747-5333-1-3

**Published:** 2006-03-13

**Authors:** William R Hersh, Ravi Teja Bhupatiraju, Laura Ross, Phoebe Roberts, Aaron M Cohen, Dale F Kraemer

**Affiliations:** 1 Oregon Health & Science University, Portland, OR, USA; 2 Biogen Idec Corp., Cambridge, MA, USA

## Abstract

**Background:**

The goal of the TREC Genomics Track is to improve information retrieval in the area of genomics by creating test collections that will allow researchers to improve and better understand failures of their systems. The 2004 track included an ad hoc retrieval task, simulating use of a search engine to obtain documents about biomedical topics. This paper describes the Genomics Track of the Text Retrieval Conference (TREC) 2004, a forum for evaluation of IR research systems, where retrieval in the genomics domain has recently begun to be assessed.

**Results:**

A total of 27 research groups submitted 47 different runs. The most effective runs, as measured by the primary evaluation measure of mean average precision (MAP), used a combination of domain-specific and general techniques. The best MAP obtained by any run was 0.4075. Techniques that expanded queries with gene name lists as well as words from related articles had the best efficacy. However, many runs performed more poorly than a simple baseline run, indicating that careful selection of system features is essential.

**Conclusion:**

Various approaches to ad hoc retrieval provide a diversity of efficacy. The TREC Genomics Track and its test collection resources provide tools that allow improvement in information retrieval systems.

## Background

The growing amount of scientific research in genomics and related biomedical disciplines has led to a corresponding growth in the amount of on-line data and information, including scientific literature. A growing challenge for biomedical researchers is how to access and manage this ever-increasing quantity of information. A recent bioinformatics textbook notes, "Few areas of biological research call for a broader background in biology than the modern approach to genetics. This background is tested to the extreme in the selection of candidate genes for involvement with a disease process... Literature is the most powerful resource to support this process, but it is also the most complex and confounding data source to search" [1].

This situation presents opportunities and challenges for the information retrieval (IR) field. IR is the discipline concerned with the indexing and retrieval of information. While it has historically focused most of its research on textual documents, the field has expanded in recent years with the growth of new information needs (e.g., question-answering, cross-language), data types (e.g., sequence data, video) and platforms (e.g., the Web) [2]. An accompanying tutorial describes the basic terms and concepts of IR [3].

### Biomedical motivations

With the advent of new technologies for sequencing the genome and proteome, along with other tools for identifying the expression of genes, structures of proteins, and so forth, the face of biological research has become increasingly data-intensive, creating great challenges for scientists who formerly dealt with relatively modest amounts of data in their research. The growth of biological data has resulted in a correspondingly large increase in scientific knowledge in what biologists sometimes call the *bibliome *or literature of biology. A great number of biological information resources have become available in recent years [4].

Probably the most important of these are from the National Center for Biotechnology Information (NCBI), a division of the National Library of Medicine (NLM) that maintains most of the NLM's genomics-related databases [5]. As IR has historically focused on text-based data, the NCBI resources of most interest to the IR community include MEDLINE (the bibliographic database of medical literature, accessed by PubMed and other systems) and textbooks such as Online Mendelian Inheritance in Man (OMIM). However, recognizing that literature is often a starting point for data exploration, there is also great interest in resources such as Entrez Gene [6], which serves as a switchboard to integrate gene information as well as provide annotation of its function using the widely accepted GeneOntology (GO) [7]. PubMed also provides linkages to full-text journal articles on the Web sites of publishers. Additional genomics resources exist beyond the NCBI, such as the model organism genome databases [8]. As with the NCBI resources, these resources provide rich linkage and annotation.

**Figure 1:**
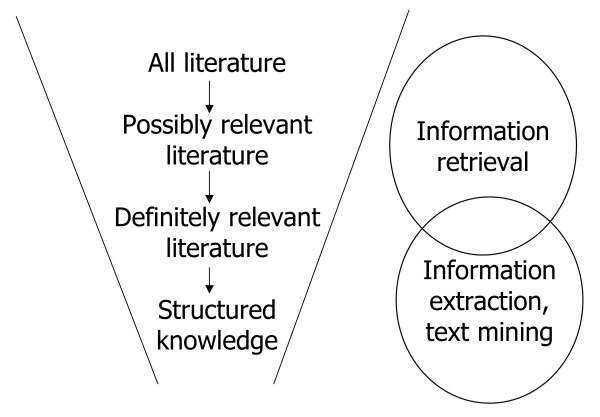
Steps in deriving knowledge from the biomedical literature and related application areas. This figure depicts the "funnel" of literature that occurs when a user seeks information and knowledge. The related information applications are shown to the right. The step of going from the entire literature to possibly relevant references is usually performed by an information retrieval system, whereas the step of identifying definitely relevant references and knowledge within them is the task of an information extraction system (or a person, since the state of information extraction is less developed than information retrieval).

Because of the growing size and complexity of the biomedical literature, there is increasing effort devoted to structuring knowledge in databases. The use of these databases has become pervasive due to the growth of the Internet and Web as well as a commitment of the research community to put as much data as possible into the public domain. Figure 1 depicts the overall process of "funneling" the literature towards structured knowledge, showing the information system tasks used at different levels along the way. This figure shows our view of the optimal uses for IR and the related areas of information extraction and text mining.

Both the IR and bioinformatics communities have long histories of forums for evaluation of methods. The latter has the well-known Critical Assessment of Methods of Protein Structure Prediction (CASP) initiative for protein structure prediction [9,10]. More recently, challenge evaluations have been initiated for researchers interested in information extraction (IE) [11], including the Knowledge Discovery from Databases (KDD) Cup [12] and the BioCreAtIvE initiative [13].

### Text retrieval conference

The IR community has had an evaluation forum in the Text Retrieval Conference (TREC, trec.nist.gov) since 1992. TREC is an annual activity of the IR research community sponsored by the National Institute for Standards and Technology (NIST) that aims to provide a forum for evaluation of IR systems and users [14]. A key feature of TREC is that research groups work on a common source of data and a common set of queries or tasks. The goal is to allow comparisons across systems and approaches in a research-oriented, collegial manner. TREC activity is organized into "tracks" of common interest, such as question-answering, multi-lingual IR, Web searching, and interactive retrieval. TREC generally works on an annual cycle, with data distributed in the spring, experiments run in the summer, and the results presented at the annual conference that usually takes place in November.

Evaluation in TREC is based on the "Cranfield paradigm" that measures system success based on quantities of relevant documents retrieved, in particular the metrics of recall and precision [2]. Operationally, recall and precision are calculated using a test collection of known documents, topics, and judgments of relevance between them. In most TREC tracks, the two are combined into a single measure of performance, mean average precision (MAP). The first step in determining MAP is to calculate the average precision of each topic, which is measured by the average of precision after each relevant document is retrieved. The mean over all of these average precision values is the MAP.

### TREC Genomics Track

The goal of the TREC Genomics Track is to create test collections for evaluation of information retrieval (IR) and related tasks in the genomics domain. The Genomics Track differs from all other TREC tracks in that it is focused on retrieval in a specific domain as opposed to general retrieval tasks, such as Web searching or question answering. The 2004 track was the second year of the TREC Genomics Track. This year was different from the first year, as we had resources available to us from a National Science Foundation (NSF) Information Technology Research (ITR) grant that allowed for programming support and relevance judgments. In contrast, for the 2003 track we had to rely on proxies for relevance judgments and other gold standard data [15]. The Genomics Track is overseen by a steering committee of individuals with a background in IR and/or genomics.

The TREC 2004 Genomics Track consisted of two tasks. The first task was a standard ad hoc retrieval task using topics obtained from real biomedical research scientists and documents from a large subset of the MEDLINE bibliographic database. The second task focused on categorization of full-text documents, simulating the task of curators of the Mouse Genome Informatics (MGI) system and consisting of three subtasks. The second task is described in a companion paper [16]. A total of 33 groups participated in the 2004 Genomics Track, making it the track with the most participants in all of TREC 2004. The remainder of this paper describes the methods and results from the ad hoc retrieval task, expanding upon the original report from the conference proceedings [17].

## Methods

The goal of the ad hoc task was to mimic conventional searching. The use case was a scientist with a specific information need, searching the MEDLINE bibliographic database to find relevant articles to retrieve.

### Documents

The document collection for the ad hoc retrieval task was a 10-year subset of MEDLINE. We contemplated the use of full-text documents in this task but were unable to procure an adequate amount to represent real-world searching. Therefore, we chose to use MEDLINE. As noted above, however, despite the widespread availability of on-line full-text scientific journals, at present most searchers of the biomedical literature still use MEDLINE as an entry point. Consequently, there is great value in being able to search MEDLINE effectively.

The subset of MEDLINE used for the track consisted of 10 years of completed citations from the database inclusive from 1994 to 2003. Records were extracted using the Date Completed (DCOM) field for all references in the range of 19940101 – 20031231. This provided a total of 4,591,008 records. We used the DCOM field and not the Date Published (DP). As a result, some records were published but not completed prior to 1994, i.e., the collection had:

• 2,814 (0.06%) DPs prior to 1980

• 8,388 (0.18%) DPs prior to 1990

• 138,384 (3.01%) DPs prior to 1994

The remaining 4,452,624 (96.99%) DPs were within the 10 year period of 1994–2004.

The data was made available in two formats:

• MEDLINE – the standard NLM format in ASCII text with fields indicated and delimited by 2–4 character abbreviations (uncompressed – 9,587,370,116 bytes, gzipped – 2,797,589,659 bytes)

• XML – the newer NLM XML format (uncompressed – 20,567,278,551 bytes, gzipped – 3,030,576,659 bytes)

### Topics

The topics for the ad hoc retrieval task were developed from the information needs of real biologists and modified as little as possible to create needs statements with a reasonable estimated amount of relevant articles (i.e., more than zero but less than one thousand). The information needs capture began with interviews by 12 volunteers who sought biologists in their local environments. A total of 43 interviews yielded 74 information needs. Some of these volunteers, as well as an additional four individuals, created topics in the proposed format from the original interview data.

We aimed to have each information need reviewed more than once but were only able to do this with some, ending up with a total of 91 draft topics. The same individuals then were assigned different draft topics for searching on PubMed so they could be modified to generate final topics with a reasonable number of relevant articles. The track chair made one last pass to make the formatting consistent and extract the 50 that seemed most suitable as topics for the track.

The topics were formatted in XML and had the following fields:

• ID – 1 to 50

• Title – abbreviated statement of information need

• Information need – full statement information need

• Context – background information to place information need in context

We created an additional five "sample" topics, e.g., topic 51:

<TOPIC>

<ID>51</ID>

<TITLE>pBR322 used as a gene vector</TITLE>

<NEED>Find information about base sequences and restriction maps in plasmids that are used as gene vectors.</NEED>

<CONTEXT>The researcher would like to manipulate the plasmid by removing a particular gene and needs the original base sequence or restriction map information of the plasmid.</CONTEXT>

</TOPIC>

### Relevance judgments

Relevance judgments were done using the conventional "pooling method" whereby a fixed number of top-ranking documents from each official run were pooled and provided to an individual (blinded to the number of groups who retrieved the document and what their search statements were). The relevance assessor then judged each document for the specific topic query as definitely relevant (DR), possibly relevant (PR), or not relevant (NR). For the official results, which required binary relevance judgments, documents that were rated DR or PR were considered relevant.

The pools were built as follows. Each of the 27 groups designated a top-precedence run that would be used for relevance judgments, typically what they thought would be their best-performing run. We took, on average, the top 75 documents for each topic from these 27 runs and eliminated the duplicates to create a single pool for each topic. The average pool size (average number of documents judged per topic) was 976, with a range of 476–1450.

The relevance judgments were done by two individuals with backgrounds in biology. One was a PhD biologist and the other an undergraduate biology student. Each topic was judged fully by one of the judges. In addition, to assess interjudge agreement, we selected every tenth article in the pool from six topics for duplicate judgment, allowing calculation of the kappa statistic for chance-corrected agreement [18].

### Evaluation measures

The primary evaluation measure for the task was mean average precision (MAP). Results were calculated using the trec_eval program, a standard scoring system for TREC. A statistical analysis was performed using a repeated measures analysis of variance, with posthoc Tukey tests for pairwise comparisons. In addition to analyzing MAP, we also assessed precision at 10 and 100 documents.

## Results

A total of 27 research groups submitted 47 different runs. Table 1 shows the pool size, number of relevant documents, mean average precision (MAP), average precision at 10 documents, and average precision at 100 documents for each topic. (Precision at 100 documents is potentially compromised due to a number of topics having many fewer than 100 relevant documents and thus being unable to score well with this measure no matter how effective they were at ranking relevant documents at the topic of list. However, as noted in Table 1, the mean and median number of relevant documents for all topics was over 100 and, as such, all runs would be affected by this issue.)

**Table 1:** Topics and associated data. Ad hoc retrieval topics, number of relevant documents, and average results for all runs.

Topic	Pool	Definitely Relevant	Possibly Relevant	Not Relevant	Definitely & Possibly Relevant	MAP average	P@10 average	P@100 average
1	879	38	41	800	79	0.3073	0.7383	0.2891
2	1264	40	61	1163	101	0.0579	0.2787	0.1166
3	1189	149	32	1008	181	0.0950	0.3298	0.2040
4	1170	12	18	1140	30	0.0298	0.0894	0.0360
5	1171	5	19	1147	24	0.0564	0.1340	0.0349
6	787	41	53	693	94	0.3993	0.8468	0.3938
7	730	56	59	615	115	0.2006	0.4936	0.2704
8	938	76	85	777	161	0.0975	0.3872	0.2094
9	593	103	12	478	115	0.6114	0.7957	0.6196
10	1126	3	1	1122	4	0.5811	0.2532	0.0277
11	742	87	24	631	111	0.3269	0.5894	0.3843
12	810	166	90	554	256	0.4225	0.7234	0.5866
13	1118	5	19	1094	24	0.0288	0.1021	0.0274
14	948	13	8	927	21	0.0479	0.0894	0.0270
15	1111	50	40	1021	90	0.1388	0.2915	0.1800
16	1078	94	53	931	147	0.1926	0.4489	0.2883
17	1150	2	1	1147	3	0.0885	0.0511	0.0115
18	1392	0	1	1391	1	0.6254	0.0660	0.0072
19	1135	0	1	1134	1	0.1594	0.0362	0.0062
20	814	55	61	698	116	0.1466	0.3957	0.2238
21	676	26	54	596	80	0.2671	0.4702	0.2796
22	1085	125	85	875	210	0.1354	0.4234	0.2709
23	915	137	21	757	158	0.1835	0.3745	0.2747
24	952	7	19	926	26	0.5970	0.7468	0.1685
25	1142	6	26	1110	32	0.0331	0.1000	0.0330
26	792	35	12	745	47	0.4401	0.7298	0.2411
27	755	19	10	726	29	0.2640	0.4319	0.1355
28	836	6	7	823	13	0.2031	0.2532	0.0643
29	756	33	10	713	43	0.1352	0.1809	0.1515
30	1082	101	64	917	165	0.2116	0.4872	0.3113
31	877	0	138	739	138	0.0956	0.2489	0.2072
32	1107	441	55	611	496	0.1804	0.6085	0.4787
33	812	30	34	748	64	0.1396	0.2234	0.1647
34	778	1	30	747	31	0.0644	0.0830	0.0668
35	717	253	18	446	271	0.3481	0.8213	0.6528
36	676	164	90	422	254	0.4887	0.7638	0.6700
37	476	138	11	327	149	0.5345	0.7426	0.6564
38	1165	334	89	742	423	0.1400	0.5915	0.4043
39	1350	146	171	1033	317	0.0984	0.3936	0.2689
40	1168	134	143	891	277	0.1080	0.3936	0.2796
41	880	333	249	298	582	0.3356	0.6766	0.6521
42	1005	191	506	308	697	0.1587	0.6596	0.5702
43	739	25	170	544	195	0.1185	0.6915	0.2553
44	1224	485	164	575	649	0.1323	0.6149	0.4632
45	1139	108	48	983	156	0.0286	0.1574	0.0711
46	742	111	86	545	197	0.2630	0.7362	0.4981
47	1450	81	284	1085	365	0.0673	0.3149	0.2355
48	1121	53	102	966	155	0.1712	0.4021	0.2557
49	1100	32	41	1027	73	0.2279	0.5404	0.2049
50	1091	79	223	789	302	0.0731	0.3447	0.2534
Mean	975.1	92.6	72.8	809.7	165.4	0.2171	0.4269	0.2637
Median	978.5	54	44.5	783	115.5	0.1590	0.3989	0.2472
Min	476	0	1	298	1	0.0286	0.0362	0.0062
Max	1450	485	506	1391	697	0.6254	0.8468	0.6700

The results of the duplicate judgments for the kappa statistic are shown in Table 2. The resulting value of kappa was 0.51, indicating a "fair" level of agreement but not being too different from similar relevance judgment activities in other domains, e.g., [19]. In general, the PhD biologist assigned more articles in the relevant category than the undergraduate.

**Table 2:** Kappa results. Kappa results for inter-judge agreement in relevant judgments for ad hoc retrieval task.

Judge 2	Definitely relevant	Possibly relevant	Not relevant	Total
Judge 1				
Definitely relevant	62	35	8	105
Possibly relevant	11	11	5	27
Not relevant	14	57	456	527
Total	87	103	469	659

The results of all participating groups are shown in Table 3. The statistical analysis for MAP demonstrated significance across all the runs, with the pair-wise significance for the top run (pllsgen4a2) not obtained until the run RMITa about one-quarter of the way down the results.

**Table 3:** Ad hoc retrieval results. All runs, sorted by mean average precision.

**Run**	**Group (reference)**	**Manual/Automatic**	**Mean Average Precision**	**Relevant at 10 documents**	**Relevant at 100 documents**
pllsgen4a2	patolis.fujita [20]	A	0.4075	6.04	41.96
uwmtDg04tn	u.waterloo.clarke [21]	A	0.3867	6.24	42.1
pllsgen4a1	patolis.fujita [20]	A	0.3689	5.7	39.36
THUIRgen01	tsinghua.ma [22]	M	0.3435	5.82	39.24
THUIRgen02	tsinghua.ma [22]	A	0.3434	5.94	39.44
utaauto	u.tampere [24]	A	0.3324	5.02	32.26
uwmtDg04n	u.waterloo.clarke [21]	A	0.3318	5.68	36.84
PSE	german.u.cairo [35]	A	0.3308	5.86	36.66
tnog3	tno.kraaij [36]	A	0.3247	5.6	36.56
tnog2	tno.kraaij [36]	A	0.3196	5.62	36.04
utamanu	u.tampere [24]	M	0.3128	6.52	38.88
aliasiBase	alias-i [23]	A	0.3094	5.38	34.58
ConversManu	converspeech [37]	M	0.2931	5.82	37.18
RMITa	rmit.scholer [38]	A	0.2796	5.12	31.4
aliasiTerms	alias-i [23]	A	0.2656	4.8	30.3
akoike	u.tokyo (none)	M	0.2427	4.48	31.3
OHSUNeeds	ohsu.hersh [25]	A	0.2343	3.84	26.46
tgnSplit	tarragon [39]	A	0.2319	4.86	29.26
UIowaGN1	u.iowa [40]	A	0.2316	4.76	28.5
tq0	nlm.umd.ul [28]	A	0.2277	5.12	30.1
OHSUAll	ohsu.hersh [25]	A	0.2272	4.32	27.76
LHCUMDSE	nlm.umd.ul [28]	A	0.2191	3.9	24.18
akoyama	u.tokyo (none)	M	0.2155	4.52	25.62
PDTNsmp4	u.padova [41]	A	0.2074	4.56	23.18
PD50501	u.padova [41]	A	0.2059	4.42	25.18
RMITb	rmit.scholer [38]	A	0.2059	4.56	27.26
UBgtNormJM1	suny.buffalo [42]	A	0.2043	4.34	25.38
ConversAuto	converspeech [37]	A	0.2013	3.88	22.8
york04g2	york.u [43]	M	0.2011	5.5	25.8
tgnNecaux	tarragon [39]	A	0.1951	4.08	23.58
lga1	indiana.u.seki [26]	A	0.1833	3.08	22.86
york04g1	york.u [43]	A	0.1794	4.14	26.96
lga2	indiana.u.seki [26]	A	0.1754	3.1	20.22
rutgersGAH1	rutgers.dayanik [44]	A	0.1702	4.66	26.76
wdvqlxa1	indiana.u.yang [45]	A	0.1582	4.2	24.78
wdvqlx1	indiana.u.yang [45]	A	0.1569	4.26	24.26
DCUmatn1	dubblincity.u [46]	M	0.1388	3.28	17.84
BioTextAdHoc	u.cberkeley.hearst [27]	A	0.1384	3.76	23.76
shefauto2	u.sheffield.gaizauskas [47]	A	0.1304	3.66	18.5
rutgersGAH2	rutgers.dayanik [44]	A	0.1303	3.42	19.48
shefauto1	u.sheffield.gaizauskas [47]	A	0.1294	3.54	18.92
run1	utwente (none)	M	0.1176	1.5	10.5
MeijiHilG	meiji.u [48]	A	0.0924	2.1	15.24
DCUma	dubblincity.u [46]	M	0.0895	2.4	15.46
csusm	u.sanmarcos [49]	M	0.0123	0.44	1.6
edinauto2	u.edinburgh.sinclair [50]	A	0.0017	0.46	1.6
edinauto5	u.edinburgh.sinclair [50]	A	0.0012	0.36	1.3
Mean			0.2074	4.48	26.46

The best official run was achieved by Patolis Corp., with a MAP of 0.4075. [20]. This run used a combination of Okapi weighting (BM25 for term frequency but with standard inverse document frequency), Porter stemming, expansion of symbols by LocusLink and MeSH records, blind relevance feedback (also known as blind query expansion), and use of all three fields of the topic (title, need, and context). This group also reported a post-submission run that added the language modelling technique of Dirichlet-Prior smoothing to achieve an even higher MAP of 0.4264. (See accompanying paper by Zhou et al. for definition of some of these terms. [3])

The next best run was achieved by the University of Waterloo [21]. This group used a variety of approaches including Okapi weighting, blind relevance feedback, and various forms of domain-specific query expansion. Their blind relevance feedback made use of usual document feedback as well as feedback from passages. Their domain-specific query expansion included expanding lexical variants as well as expanding acronym, gene, and protein name synonyms.

A number of groups used boosting of word weights in queries or documents. Tsinghua University boosted words in titles and abstracts, along with using blind query expansion [22]. Alias-i Corp. boosted query words in the title and need statements [23]. University of Tampere found value in identifying and using bi-gram phrases [24].

**Figure 2:**
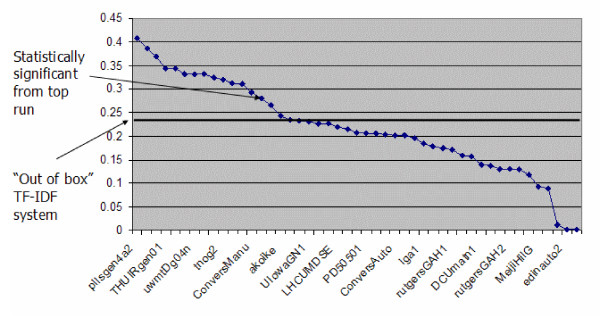
Ad hoc retrieval runs sorted by MAP. This figure shows all of the runs (x-axis) sorted by MAP (y-axis). The highest run to obtain statistical significance (RMITa) from the top run (pllsgen4a2) is denoted, along with the "out of the box" TF*IDF run (OHSUNeeds) are annotated. (Only every fifth run identifier is shown to make the x-axis more readable.)

A number of groups implemented techniques, however, that were detrimental. This is evidenced by the OHSU runs, which used the Lucene system "out of the box" that applies TF*IDF weighting [25]. Approaches that attempted to map to controlled vocabulary terms did not fare as well, such as Indiana University [26], University of California Berkeley [27], and the National Library of Medicine [28]. Many groups tried a variety of approaches, beneficial or otherwise, but usually without comparing common baseline or running exhaustive experiments, making it difficult to discern exactly which techniques provided benefit. Figure 2 shows the official results graphically with annotations for the first run statistically significant from the top run as well as the OHSU "baseline."

**Figure 3:**
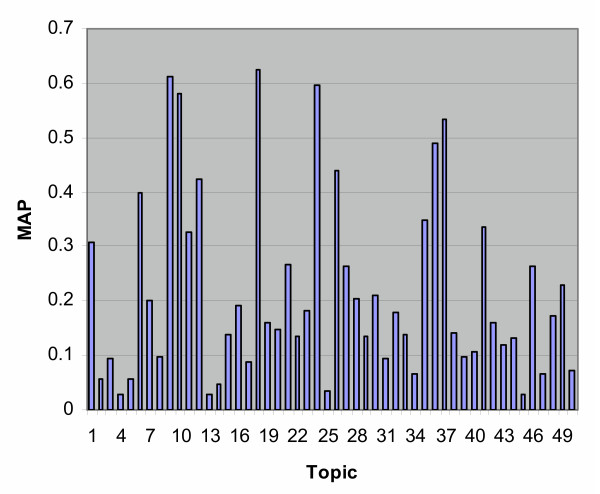
MAP by topic for the ad hoc task.

**Figure 4:**
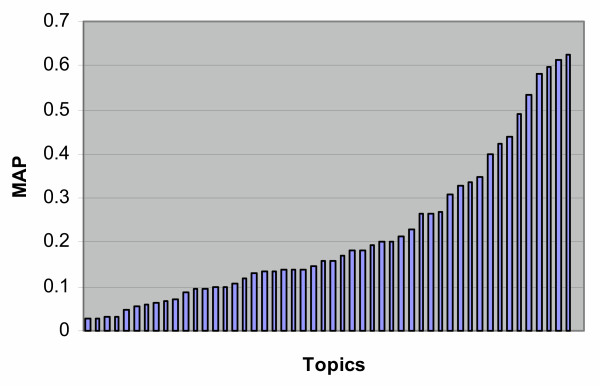
MAP by topic for the ad hoc task sorted by MAP.

**Figure 5:**
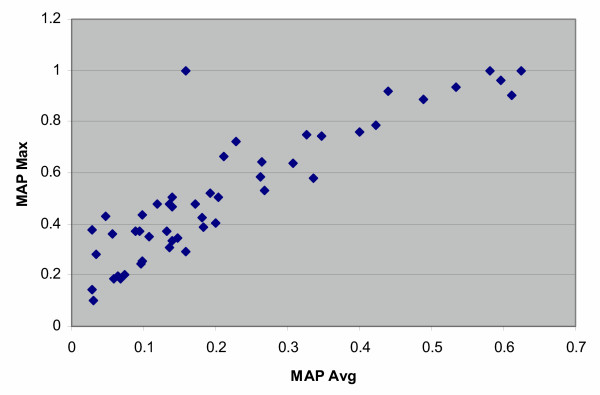
The maximum MAP plotted vs. average MAP for the ad hoc retrieval task runs.

**Figure 6:**
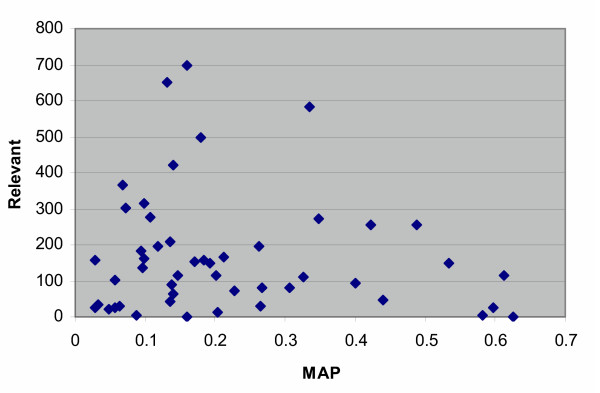
The number of relevant per topic plotted vs. MAP for the ad hoc retrieval task

As typically occurs in TREC ad hoc runs, there was a great deal of variation within individual topics, as is seen in Table 1. Figure 3 shows the average MAP across groups for each topic. Figure 4 presents the same data sorted to give a better indication of the variation across topics. There was a fairly strong relationship between the average and maximum MAP for each topic (Figure 5), while the number of relevant per topic versus MAP was less associated (Figure 6).

## Discussion

The TREC 2004 Genomics Track was very successful, with a great deal of enthusiastic participation. In all of the tasks, a diversity of approaches were used, resulting in wide variation across the results. Trying to discern the relative value of them is challenging, since few groups performed parameterized experiments or used common baselines.

In the ad hoc retrieval task, the best approaches employed techniques known to be effective in non-biomedical TREC IR tasks. These included Okapi weighting, blind relevance feedback, and language modelling. However, some domain-specific approaches appeared to be beneficial, such as expanding queries with synonyms from controlled vocabularies that are widely available. There also appeared to be some benefit for boosting parts of the queries. However, it was also easy for many groups to do detrimental things, as evidenced by the OHSU run of a TF*IDF system "out of the box" that scored well above the median.

How well do systems in the Genomics Track, i.e., systems focused on IR in the genomics domain, perform relative to systems in other domains? This is of course a challenging question to answer, since differing results may not only be due to different systems, but also different test collections, topics, and/or relevance judgments. The most comprehensive analysis of this issue to date has come from Buckley and Voorhees, who compared various yearly tasks and best performing systems with the general TREC ad hoc task data [29]. Tasks provided with greater topic elaboration performed better (MAP around 0.35–0.40) than those with shorter topics. The Genomics Track topics could be considered comparable to these topics, with comparable results. It has been noted that TREC tracks with far larger document collections, e.g., the Terabyte Track [30] and the Web Track [31], achieve much lower best MAP scores, with none better than 0.28. Although we did not address this issue explicitly, the data obtained through the experiments of the Genomics Track should allow further investigation of attributes that make genomics IR harder or easier than other IR task domains.

This work, and IR evaluation using test collections generally, have a number of methodological limitations. Generally, evaluation using test collections is more appropriate for evaluating IR systems than such systems in the hands of real users. TREC does have a small history of interactive IR evaluation [32], with the results showing that successful use of the system is not necessarily associated with better recall and precision [33].

Another limitation of evaluation using test collections is inconsistency of relevance judgments. This problem is well-known in the construction of test collections [19], but research has generally shown that using different judgments affects absolute but not relative performance [34]. In other words, different judgments lead to different MAP and other scores, but systems that perform well with one set of judgments tend to do as relatively well with others. Unfortunately we did not perform enough duplicate judgments to assess the impact of different judgments in the 2004 track. We will aim to perform this analysis in future offerings of the track.

Despite these limitations, the test collection and the results obtained provide substantial data for further research. A variety of additional issues can be investigated, such as attributes of documents and topics (including linguistics aspects like words and concepts present or absent) that are associated with relevance. In addition, a 2005 offering of the Genomics Track will take place, providing additional data for further research.

## Conclusion

The ad hoc retrieval task of the TREC Genomics Track has developed resources that allow researchers to assess systems and algorithms for search in the genomics domain. The data for the 2004 track has been released to the general community for continued experimentation, and further annual offerings of the track will enhance these tools. The lessons learned from the 2004 track will guide both the operation and research of future offerings of the track in 2005 and beyond.

## Competing interests

The author(s) declare that they have no competing interests.

## Authors' contributions

WRH conceptualized the TREC Genomics Track, directed the organization of the ad hoc retrieval task, and drafted this paper. RTB did all of programming and collation of data. LR and PR performed the relevance judgments. AMC provided feedback and critical insights with the running of the track and editing of the paper. DFK performed the statistical analyses.
